# The Effect of Continuous Liver Normothermic Machine Perfusion on the Severity of Histological Bile Duct Injury

**DOI:** 10.3389/ti.2023.11645

**Published:** 2023-09-04

**Authors:** Nicholas Gilbo, Desley Neil, Rebecca Brais, Steffen Fieuws, Letizia Lo Faro, Peter Friend, Rutger Ploeg, Diethard Monbaliu

**Affiliations:** ^1^ Laboratory of Abdominal Transplantation, Department of Microbiology, Immunology and Transplantation, Faculty of Medicine, KU Leuven, Leuven, Belgium; ^2^ University Hospital of Liège, Liège, Belgium; ^3^ Department of Cellular Pathology, Queen Elizabeth Hospital, Birmingham, United Kingdom; ^4^ Department of Pathology, Cambridge University Hospitals NHS Foundation Trust, Cambridge, United Kingdom; ^5^ Interuniversity Center for Biostatistics and Statistical Bioinformatics, UZ KU Leuven, Leuven, Belgium; ^6^ Oxford Transplant Centre, Nuffield Department of Surgical Sciences, University of Oxford, Oxford, United Kingdom; ^7^ Transplantation Research Group, Department of Microbiology, Immunology and Transplantation, KU Leuven, Leuven, Belgium

**Keywords:** liver transplantation, normothermic machine perfusion, ischemic cholangiopathy, bile duct injury, liver viability assessment

## Abstract

Static Cold Storage (SCS) injures the bile duct, while the effect of Normothermic Machine Perfusion (NMP) is unknown. In a sub-study of the COPE trial on liver NMP, we investigated the impact of preservation type on histological bile duct injury score (BDIS). Transplants with at least one bile duct biopsy, either at end of preservation or 1 h post-reperfusion, were considered. BDIS was determined by assessing peribiliary glands injury, stromal and mural loss, haemorrhage, and thrombosis. A bivariate linear model compared BDIS (estimate, CI) between groups. Sixty-five transplants and 85 biopsies were analysed. Twenty-three grafts were preserved with SCS and 42 with NMP, with comparable baseline characteristics except for a shorter cold ischemic time in NMP. The BDIS increased over time regardless of preservation type (*p* = 0.04). The BDIS estimate was higher in NMP [8.02 (7.40–8.65)] than in SCS [5.39 (4.52–6.26), *p* < 0.0001] regardless of time. One patient in each group developed ischemic cholangiopathy, with a BDIS of 6 for the NMP-preserved liver. In six other NMP grafts, BDIS ranged 7–12 without development of ischemic cholangiopathy. In conclusion, BDIS increases over time, and the higher BDIS in NMP did not increase ischemic cholangiopathy. Thus, BDIS may overestimate this risk after liver NMP.

## Graphical Abstract

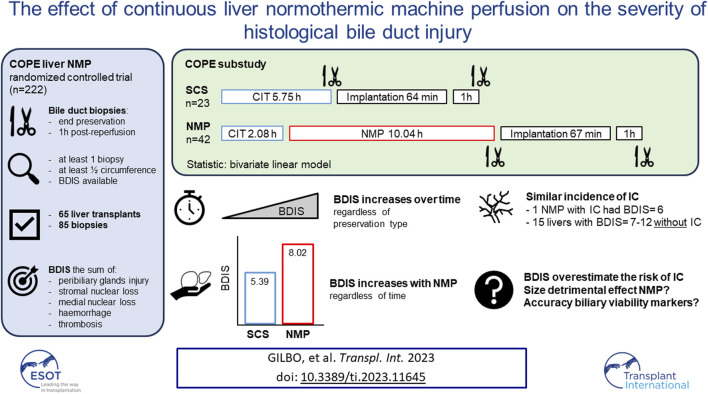

## Introduction

Biliary complications are a significant cause of morbidity after liver transplantation, particularly in the form of non-anastomotic strictures of the biliary tree, also referred to as ischemic cholangiopathy (IC) [1].

The pathogenesis of IC involves ischemic-reperfusion injury, immune-mediated damage, cytotoxic insults, and defective biliary regeneration [1]. Additionally, livers procured from donation after circulatory death (DCD) donors [2] are more prone to developing IC than those procured from donation after brain death (DBD) donors, due to the additional hit of warm ischemic injury during the donation process [1]. Static cold storage (SCS) is inadequate in maintaining the integrity of the biliary epithelium, leading to epithelial loss at the end of SCS in up to 86% of livers [3]. After reperfusion, this injury progresses, affecting the peribiliary vascular space [4] and damaging the stem cell niche of the peribiliary glands [5]. Histological injuries such as epithelial loss, mural stroma necrosis, intramural haemorrhage, peribiliary vascular injury, thrombosis, and loss of peribiliary glands, have been identified as predictors of IC [5]. This formed the basis for the histological bile duct injury score (BDIS) [4–6], which has been used to stratify the risk of IC.

To address the need for improved preservation and reduce complications after transplantation of high-risk livers [7], alternative preservation methods, like continuous liver normothermic machine perfusion (NMP), have gained interest. Liver NMP involves the *ex situ* perfusion of the graft with an oxygenated, nutrient-enriched, erythrocytes-based perfusate kept at 37°C [8]. Two randomized controlled trials demonstrated that continuous NMP reduces ischemic-reperfusion injury (as measured by post-transplant transaminase release) of low to intermediate-risk livers [9, 10]. Although in a porcine DCD model continuous NMP has shown promising results in preserving the histology and promoting biliary regeneration [11], the results from currently available randomized controlled trials are inconclusive on the prevention of IC after transplantation. The consortium for organ preservation in Europe (COPE) trial on liver NMP did not find any difference in the incidence of IC between NMP and SCS, but it was not powered for this research question [9]. In contrast, Markmann et al. showed a significant reduction in the incidence of ischemic biliary complications, which were however defined as biliary strictures or leakage [10]. Furthermore, in early clinical series on end-ischemic NMP [12, 13] up to 30% of liver grafts transplanted after NMP viability assessment based on perfusate biochemistry [14] developed IC, suggesting that NMP does not prevent biliary injury. This prompted the definition of criteria to select livers at low risk of developing IC based on biliary biomarkers, which were identified utilizing BDIS as a surrogate endpoint for IC [6]. Although these criteria are increasingly being used in clinical practice, the impact of liver NMP on the severity of histological bile duct injury or its correlation with the development of IC remains unknown. Therefore, this study aims to investigate the influence of preservation methods on biliary injury severity, specifically utilizing the histological BDIS in a subset of liver transplants included in the COPE trial comparing liver NMP to SCS [9].

## Patients and Methods

### Study Design

**FIGURE 1:**
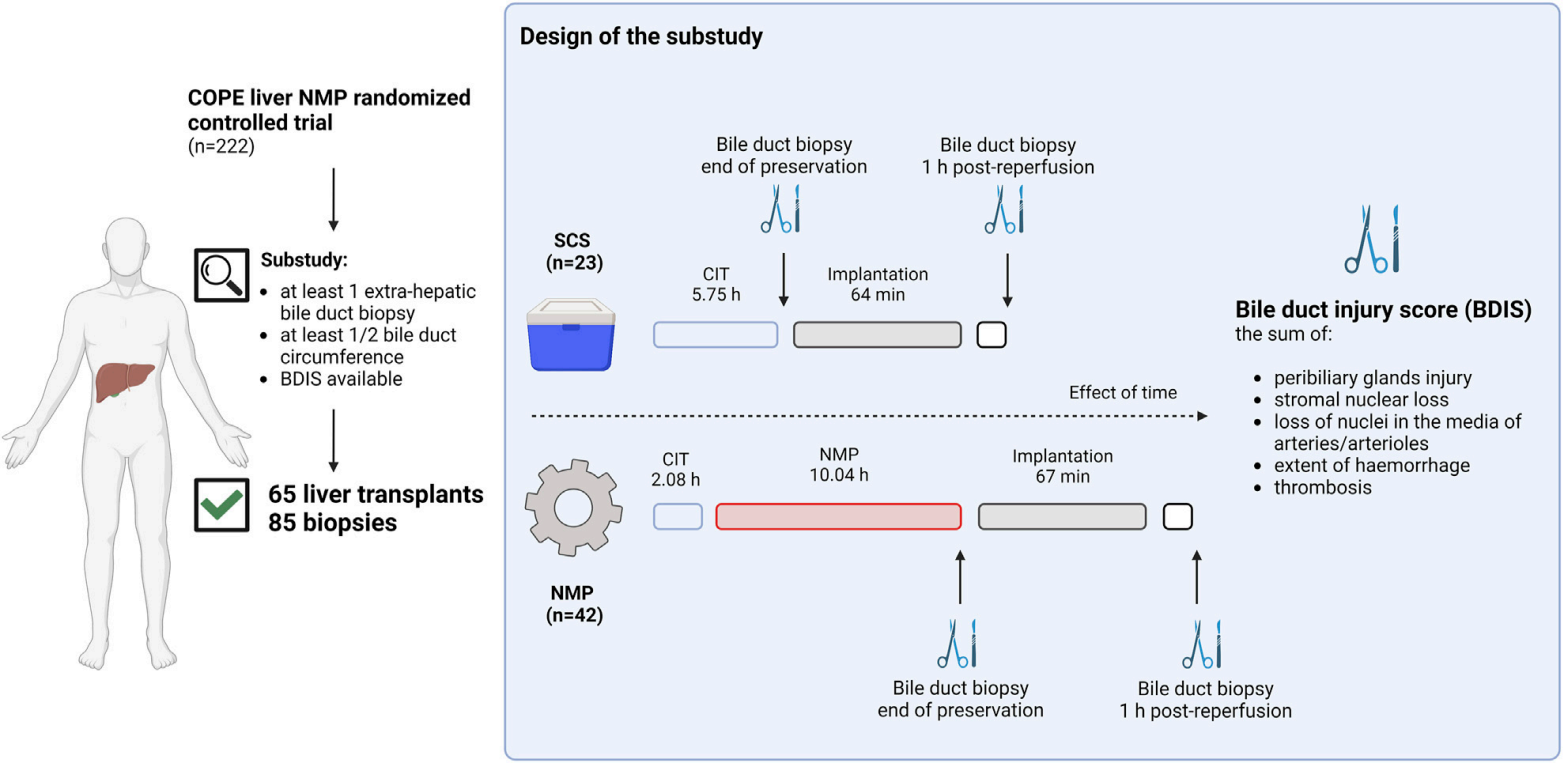
Schematic representation of the study design. Liver transplants included in the consortium for organ preservation in Europe (COPE) randomized controlled study on liver normothermic machine perfusion (NMP) with at least one biopsy of the extrahepatic bile duct consisting of at least ½ duct circumference were included in the substudy. Bile duct biopsies were collected at the end of preservation or 1 hour after reperfusion in the recipient. In liver preserved with static cold storage (SCS) biopsies at the end of preservation were taken after a median of 5.75 h from organ flush in the donor, whilst in liver preserved with NMP these were taken after a median of 12.12 h from organ flush. Two experienced pathologists blinded for group allocation scored H&E stained slides to grade the bile duct injury score (BDIS) as the sum of peribiliary glands injury, stromal nuclear loss, loss of nuclei in the media of arteries and/or arterioles, extent of haemorrhage, and thrombosis. Only complete cases where all features could be scored were considered for the final analysis. The BDIS was compared between group. Additionally, the effect of time, donor type, and other donor, recipient, transplant, and perfusion characteristics on the BDIS were also explored. Created with Biorender.com.

Figure 1 provides a visual representation of the design of this substudy of the COPE trial. The COPE multicentre randomized trial run between June 2014 and March 2016, and considered whole livers from DBD and DCD donors aged ≥16 years. Livers were randomized 1:1 to be preserved using SCS or liver NMP started at the donor’s site with the OrganOx Metra device (OrganOx Ltd., Oxford, United Kingdom). Eligible recipients were at least 18 years old and listed for a solitary liver transplant, excluding those with fulminant liver failure. Participants were consented while on the waiting list, with the consent including the recording of anonymized data on donor, recipient, transplant, and perfusion characteristics, as well as the collection of biological samples for biobanking. Transplant centres from the United Kingdom (Addenbrooke’s Hospital, Cambridge; King’s College Hospital, London; Queen Elizabeth Hospital, Birmingham; and Royal Free Hospital, London) and Belgium (University Hospitals of Leuven, Leuven) collected samples of the extrahepatic bile duct, stored in a central biobank. Bile duct biopsies were obtained at the end of liver preservation and after 1 hour of reperfusion in the recipient. Only liver transplants with at least one biopsy consisting of at least half circumference of the bile duct were considered. The study aimed to investigate the impact of preservation (SCS or NMP) on BDIS and explore interactions with donor types. Additionally, donor, recipient, and transplant characteristics influencing BDIS severity were explored. Ischemic cholangiopathy was defined as the unequivocable evidence of extra-anastomotic biliary strictures with a patent hepatic artery observed at a protocol magnetic resonance cholangiopancreatography at the 6-month post-transplantation [9]. Histological sections were assessed by experienced liver pathologists, and BDIS scores were compared between preservation groups. The substudy was approved by the Research Ethics Committee London-Dulwich, United Kingdom (ref: 14/LO/0182).

### Normothermic Machine Perfusion

The OrganOx Metra device was used for automated NMP. NMP continued until the transplant team was ready to implant the liver, with a minimum duration of 4 h and a maximum of 24 h. Details on device, perfusate composition, and perfusion settings were already reported by Nasralla et al. [9].

### Histopathology

Two experienced liver transplant pathologists (DN and RB) assessed whole slide images of formalin fixed and paraffin embedded extrahepatic bile duct biopsies. Images were scanned at ×20 magnification on a 3DHistech scanner at 0.276 µm/pixel. Assessment focused on the completeness of bile duct section (circumferential, more than half circumference, half circumference, less than half circumference, fragment only, no bile duct tissue). To minimise sampling error, only bile ducts that included at least 50% of the circumference were included, as changes vary around the circumference of the bile duct. The pathologists evaluated the slides in a blinded manner, using a modified version of the Hansen [4]—op den Dries [5] score. The scoring focused on grading injury to deep peribiliary glands (0, normal; 1, mild injury; 2, moderate injury; 3, severe injury), stromal nuclear loss (0, normal; 2, focal loss <10%; 2 moderate loss 10%–50%; 3, extensive loss >50%), loss of nuclei in the media of arteries/arterioles (0, normal; 1, focal—occasional arteries/arterioles; 2, moderate—more than occasional arteries/arterioles; 3, extensive), the extent of haemorrhage (0, none; 1, few scattered RBCs; 2, <25%; 3, 25%–50%; 4, >50%) and presence of thrombi (Y/N). The scoring excluded assessment of the epithelial lining and superficial peribiliary glands, which were not associated with IC in previous case series [5]. The pathologists underwent calibration and agreement on definitions and cut-offs before assessing the study samples. The BDIS was calculated as sum of the scores for each histological feature [6]. Only complete cases, where all features could be graded, were considered for BDIS calculation.

### Statistical Analyses

Categorical data are presented as number (percentage), while continuous variables are reported as median (IQR). Fisher exact test and Mann-Whitney U tests were used for comparing categorical and continuous characteristics, transplant data, and outcomes between the SCS and NMP groups, respectively. Kaplan-Meier estimates and log-rank tests were used to assess overall and graft survival, with cumulative incidence curves used to visualize graft loss (considering patient death without preceding graft loss as a competing event). Between-group comparisons utilized Gray’s test.

To compare BDIS between SCS and NMP, a bivariate linear model was employed, considering preservation (SCS, NMP) and donor type (DBD, DCD) as fixed factors and centre as a random factor. This model accounted for missing biopsy values and utilized an unstructured 2 × 2 covariance matrix for BDIS scores at the two timepoints. This model returned BDIS estimate (i.e., mean from a multivariate regression model for longitudinal measures) with a 95% confidence interval. The model was also used to examine changes in BDIS over time and to assess the interaction between preservation type and time. Subgroup analyses for DBD and DCD patients and exploration of the interaction between donor type and preservation type were performed. A *post hoc* sensitivity analysis included only liver transplants with increasing BDIS over time.

To evaluate the relationship between donor, recipient, and transplant characteristics with BDIS, bivariate linear models were used for each variable, allowing for differences in the linear relationship between the two timepoints. Regression coefficients were reported for each timepoint and averaged. No corrections for multiple testing were applied; therefore, these *p*-values should be interpreted as exploratory. Variables significantly associated with BDIS in the latter analyses were combined in a multivariable model. SAS software, version 9.4, was used for all analyses.

## Results

### Study Population

**FIGURE 2:**
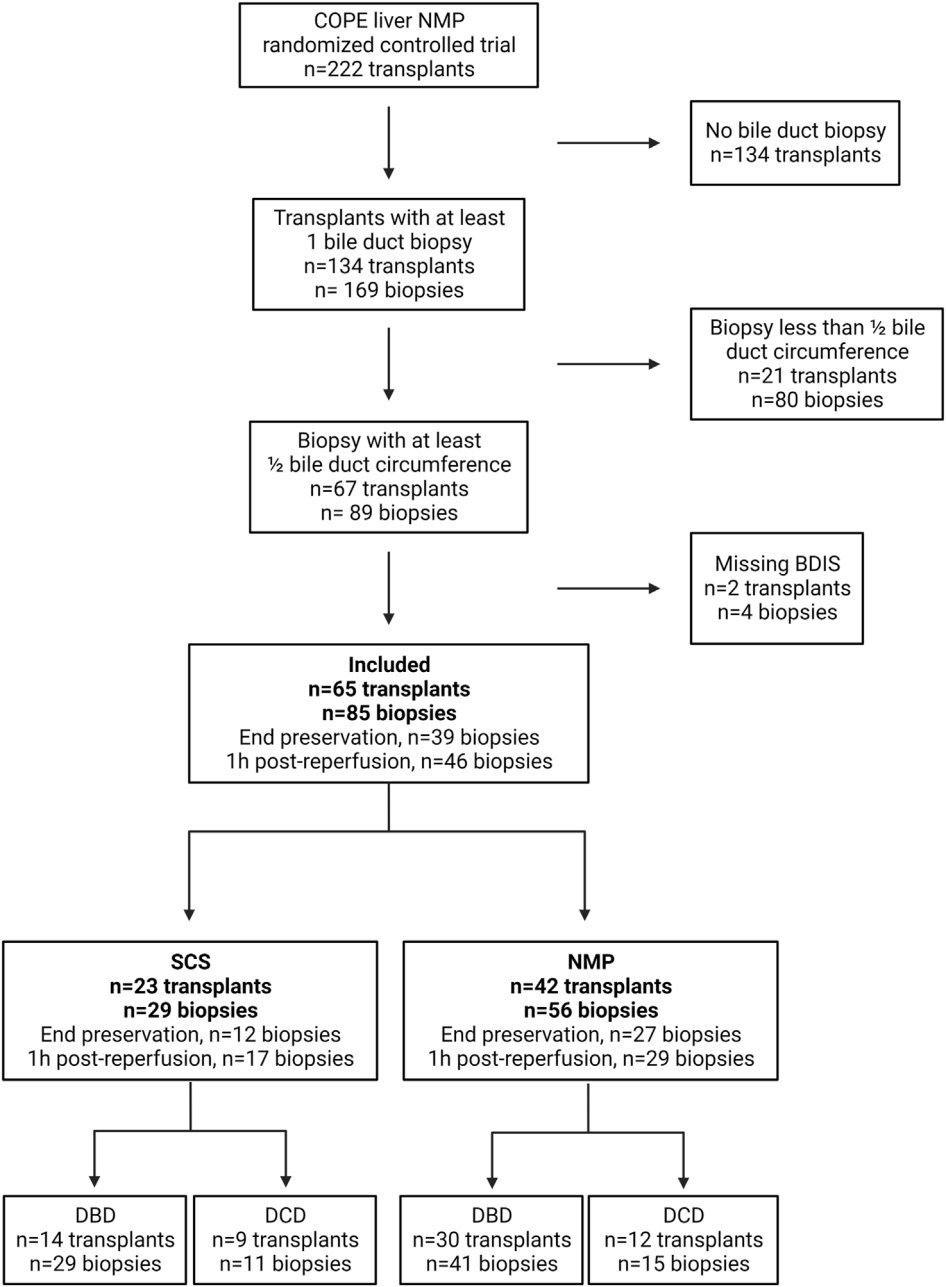
Diagram depicting patients’ inclusion in a substudy of the consortium for organ preservation in Europe (COPE) randomized controlled trial on liver normothermic machine perfusion (NMP). BDIS, bile duct injury score; DBD, donation after brain death; DCD, donation after circulatory death; SCS, static cold storage. Created with Biorender.com.

The COPE trial included 222 liver transplants. Bile duct biopsies were unavailable for 134 transplants, and an additional 21 were excluded because the biopsies consisted of <50% of bile duct circumference. Finally, four biopsies belonging to two subjects were excluded because some feature of the BDIS could not be scored due to artifacts (Figure 2). In total, 65 transplants were included in this study and 85 bile duct biopsies were evaluated. Of these, 42 livers (56 biopsies) were preserved using continuous NMP, and 23 livers (29 biopsies) were preserved using SCS. In the NMP group 30/42 (71.43%) livers were procured from DBD donors and 12/42 (28.57%) from DCD donors, whilst in the SCS group 14/23 (60.87%) livers were procured from DBD donors and 9/23 (39.13%) from DCD donors (*p* = 0.38).

The included subjects were well matched in terms of donor, recipient, and transplant characteristics. However, there were significant differences in the cause of death and the highest concentration of sodium in the donor, and in cold ischemic time [NMP: 2.17 (1.83–2.33) hours, SCS: 5.75 (5.08–7.) hours; *p* < 0.001] (Table 1). In DCD livers, donor warm ischemic time was 23.50 (21.00–28.50) minutes in the NMP group and 24 (23–27) minutes in the SCS group (*p* = 0.89). The most frequent indication for transplantation was alcoholic liver cirrhosis in both groups, and recipients were transplanted with a median lab MELD of 13 (10–18) points in the NMP group, and 11 (9–16) points in the SCS group (*p* = 0.44).

**TABLE 1:** Donor, recipient, and transplant characteristics of the study population.

	SCS (*n* = 23)	NMP (*n* = 42)	*p*-value
**Donor demographics**			
Donor Type, *n* (%)			
DBD	14 (60.87%)	30 (71.43%)	0.38
DCD	9 (39.13%)	12 (28.57%)	
Total donor warm ischemia time,^a^ min	24 (23–27)	23 (21–29)	0.89
Donor age, years	56 (47.56–62.78)	57 (45.30–72.81)	0.58
Donor gender, n (%)			
Male	14 (60.87%)	20 (47.62%)	0.31
Female	9 (39.13%)	22 (52.38%)	
Donor BMI, Kg/m^2^	24 (−27)	25 (−27)	0.57
Donor blood group, n (%)			
A	12 (52.17%)	23 (54.76%)	0.62
AB	1 (4.35%)	1 (2.38%)	
B	3 (13.04%)	2 (4.76%)	
0	7 (30.43%)	16 (38.10%)	
Donor admitted to the ICU, *n* (%)	23 (100%)	42 (100%)	—
Length of donor ICU stay, days	2 (2–6)	2 (2–4)	0.47
Donor cause of death, *n* (%)			0.04
Others	3 (13.04%)	5 (11.90%)	
Trauma	4 (17.39%)	0 (0.00%)	
Hypoxia	3 (13.04%)	10 (23.81%)	
Cerebrovascular accident	13 (56.52%)	27 (64.29%)	
DRI, points	1.77 (1.36–2.68)	1.42 (1.17–2.66)	0.37
ET-DRI, points	2.00 (1.70–2.49)	1.76 (1.53–2.11)	0.30
History of diabetes, n (%)	1 (4.35%)	5 (11.90%)	0.31
History of smoking, n (%)	11 (47.83%)	13 (30.95%)	0.18
History of alcohol consumption, n (%)	6 (26.09%)	5 (12.20%)	0.16
History of cardiac disease, n (%)	6 (28.57%)	4 (11.43%)	0.11
Vasopressors use, n (%)	14 (63.64%)	24 (57.14%)	0.62
Dopamine, n (%)	0 (0%)	3 (12.50%)	0.17
Dobutamine, n (%)	0 (0%)	2 (8.33%)	0.27
Noradrenaline, n (%)	11 (78.57%)	18 (75.00%)	0.80
Vasopressin, n (%)	9 (64.29%)	14 (58.33%)	0.72
Highest AST, IU/L	28 (22–66)	52 (29–66)	0.37
Highest ALT, IU/L	31 (19–48)	32 (19–57)	0.99
Highest GGT, IU/L	34 (22–162)	47 (25–114)	0.55
Highest bilirubin, μmol/L	8 (4–18)	9 (5–17)	0.60
Highest sodium, mEq/L	148 (142–154)	141 (138–145)	0.01
Donor hepatectomy time, minutes	43 (30–57)	31 (24–36)	<0.001
Cold ischemic time, hours	5.75 (5.08–7.23)	2.08 (1.83–2.33)	<0.001
Duration NMP, hours	—	10.04 (6.25–12.09)	
**Recipient demographics**			
Transplant centre, n (%)			0.06
Cambridge	4 (17.39%)	4 (9.52%)	
King’s College	1 (4.35%)	5 (11.90%)	
Birmingham	10 (43.48%)	29 (69.05%)	
Royal Free	5 (21.74%)	2 (4.76%)	
Leuven	3 (13.04%)	2 (4.76%)	
Recipient age, years	57.40 (52.51–62.44)	54.26 (43.01–62.66)	0.33
Recipient gender, n (%)			0.17
Male	18 (78.26%)	25 (59.52%)	
Female	5 (21.74%)	17 (40.48%)	
Recipient BMI, kg/m^2^	25 (24–29)	26 (23–31)	0.49
Recipient blood group, n (%)			0.90
A	11 (47.83%)	21 (50.00%)	
AB	1 (4.35%)	3 (7.14%)	
B	3 (13.04%)	3 (7.14%)	
0	8 (34.78%)	15 (35.71%)	
Blood group match, n (%)			1
Identical	22 (95.65%)	39 (92.86%)	
Compatible	1 (4.35%)	3 (7.14%)	
Creatinine, mmol/L	70.00 (53.00–94.00)	70.50 (55.00–93.00)	0.95
Bilirubin, μmol/L	23.00 (8.00–32.00)	30.00 (10.00–63.00)	0.19
INR	1.30 (1.20–1.70)	1.28 (1.20–1.60)	0.38
Lab MELD, points	11.00 (9.00–16.00)	13 (10.00–18.00)	0.44
Indication to transplantation, n (%)			0.75
Alcoholic cirrhosis	10 (43.48%)	12 (28.57%)	
Budd Chiari	1 (4.35%)	1 (2.38%)	
Caroli’s syndrome	0 (0.00%)	1 (2.38%)	
Hemocromatosis	4 (17.39%)	7 (16.67%)	
HAT	0 (0.00%)	2 (4.76%)	
Ornithine transcarbamylase deficiency	1 (4.35%)	0 (0.00%)	
Polycystic liver disease	1 (4.35%)	3 (7.14%)	
Biliary cirrhosis	2 (8.70%)	4 (9.52%)	
Sarcoidosis	1 (4.35%)	5 (11.90%)	
Secondary sclerosing cholangitis	3 (13.04%)	7 (16.67%)	
**Transplantation**			
Steatosis, n (%)			0.38
None	10 (43.48%)	12 (29.27%)	
Mild	10 (43.48%)	16 (39.02%)	
Moderate	2 (8.70%)	10 (24.39%)	
Severe	1 (4.35%)	3 (7.32%)	
Liver weight, g	1,461 (1,267–1,658)	1,354 (1,140–1,641)	0.66
Porto caval bypass, n (%)	7 (38.89%)	9 (22.50%)	0.50
Veno-venous bypass, n (%)	1 (5.88%)	1 (2.44%)	0.51
Vena cava anastomosis, n (%)			0.66
Cava replacement	3 (13.04%)	4 (9.52%)	
Piggyback	20 (86.96%)	38 (90.48%)	
Portal vein anastomosis time, min	33 (28–40)	32 (24–45)	0.60
Hepatic artery anastomosis time, min	32 (26–39)	34 (26–41)	0.72
Total implantation time, min	64 (59–75)	67 (50–95)	0.76
Intra-operative immunosuppression, n (%)			0.44
None	14 (60.87%)	23 (54.76%)	
Others	1 (4.35%)	0 (0.00%)	
Methylprednisolone	8 (34.78%)	18 (42.86%)	
Basiliximab + methyprednisolone + tacrolimus	0 (0.00%)	1 (2.38%)	
**Outcomes**			
Peak AST within 7 days, IU/L	741 (474–2,221)	381 (196–906)	0.01
Peak ALT within 7 days, IU/L	710 (268–1,316)	348 (173–1,044)	0.06
Peak Bilirubin, μmol/L	74 (31–136)	66 (33–128)	0.99
Peak GGT, IU/L	578 (348–870)	551 (355–751)	0.70
Peak INR	1.75 (1.40–2.10)	1.70 (1.46–1.96)	0.79
AST, IU/L			
at 7 days	41 (29–91)	57 (35–127)	0.30
at 30 days	19 (15–41)	21 (13–38)	0.77
at 6 months	21 (18–35)	26 (21–34)	0.35
ALT, IU/L			
at 7 days	89 (59–190)	128 (70–327)	0.93
at 30 days	26 (18–43)	30 (21–57)	0.23
at 6 months	20 (14–30)	16 (16–43)	0.14
Bilirubin, μmol/L			
at 7 days	29 (15–72)	36 (17–100)	0.50
at 30 days	14 (8–21)	13 (7–19)	0.37
at 6 months	8 (5–15)	9 (6–14)	0.98
GGT, IU/L			
at 7 days	328 (240–723)	525 (353–719)	0.68
at 30 days	196 (63–319)	195 (122–473)	0.32
at 6 months	30 (11–205)	61 (42–222)	0.26
INR			
at 7 days	1.1 (1–1.2)	1.12 (1–1.3)	0.50
at 30 days	1.1 (1–1.2)	1.1 (1–1.2)	0.99
at 6 months	1 (1–1.1)	1.13 (1–1.23)	0.054
Post-reperfusion syndrome,^b^ n (%)	8 (34.78%)	6 (14.29%)	0.055
Post-reperfusion mean arterial pressure, mmHg	57 (52–66)	69 (64–89)	<0.001
Post-reperfusion vasopressor, n (%)	18 (81.82%)	22 (53.66%)	0.03
Post-reperfusion lactate, mmol/L	3.60 (3.20–5.10)	3.60 (2.60–4.40)	0.46
1-year patient survival, % (95% CI)	91.30 (69.50–97.80)	95.10 (81.80–98.80)	0.65
1-year graft survival, % (95% CI)	91.30 (69.50–97.80)	90.50 (76.60–96.30)	0.83
Graft loss at 1-year, % (95% CI)	8.70 (1.40–24.60)	9.50 (3.00–20.70)	0.60

ALT, alanine transaminase; AST, aspartate transaminase; BMI, body mass index; DBD, donation after brain death; DCD, donation after circulatory arrest; DRI, donor risk index; ET-DRI, EuroTransplant donor risk index; GGT, gamma glutamyl transferase; INR, international normalized ratio; MELD, model for end-stage liver disease.

^a^ Total donor warm ischemic time in DCD, donors is measured from the withdraw of life sustaining therapy to cold flush.

^b^ Post-reperfusion syndrome was defined as > 30% drop in mean arterial pressure persisting for > 1 min within 5 min of reperfusion.

In line with the findings from the COPE trial, the peak concentration of aspartate transaminase within 7 days post-transplantation was lower in the NMP group [381 (196–906) IU/dL] than in the SCS group [741 (474–2,221) IU/dL, *p* = 0.01; Table 1]. Additionally, the mean arterial pressure after reperfusion was higher in NMP [76 (64–89) mmHg, SCS: 57 (52–66) mmHg; *p* < 0.001] and the requirement for vasopressors infusion lower [22/42 (53.66%)] than SCS-preserved livers [18/23 (81.82%), *p* = 0.03], as a result of less frequent post-reperfusion syndrome [NMP: 6/42 (14.29%), SCS: 8/23 (34.78%); *p* = 0.055]. There was no difference in 1 year patient or graft survival (Supplementary Figure S1). Two patients in the COPE trial developed IC after transplantation, one in each study arm. A bile duct biopsy was available only for the NMP-preserved liver (at 1-h post-reperfusion). Therefore, the SCS-preserved liver that develop IC was excluded from this substudy.

Excluded cases had a significantly higher donor BMI, lower EuroTransplant-donor risk index, higher donor sodium, longer cold ischemic and portal vein anastomosis time, and more frequent need for vasopressor pre-reperfusion than transplants included in this substudy (Supplementary Table S1).

### Does the Type of Liver Preservation Influence Histological Bile Duct Injury?

The severity of BDIS changed significantly over time during the transplant process, regardless of the type of preservation (*p* = 0.04; Table 2). Specifically, BDIS increased after graft reperfusion in most transplants, but in four livers (two in each group) the BDIS was found to be markedly improved after 1-h post-reperfusion compared to corresponding biopsies at the end of preservation (Supplementary Figure S2). Further re-evaluation of these four pairs revealed signs of sampling injuries. Notably, there was a pronounced loss of stromal nuclei and more severe injury to the deep peribiliary glands and arteries (Supplementary Figure S3) in biopsies at the end of preservation. This degree of stromal change is suggestive of localised clamp injury, which has artificially increased the BDIS at the end of preservation.

**TABLE 2:** Multivariate regression model for longitudinal measures estimating the effect of preservation type on the histological bile duct injury score.

BDIS	SCS (*n* = 23)	NMP (*n* = 42)		
	Estimate^a^ (CI)	Estimate^a^ (CI)	*p*-value	Bonferroni
Main effect preservation type^b^	5.39 (4.52; 6.26)	8.02 (7.40; 8.65)	<0.0001	—
End preservation	5.17 (3.96; 6.39)	7.25 (6.44; 8.06)	0.006	0.006
1 h post-LT	5.61 (4.49; 6.73)	8.80 (7.94; 9.66)	<0.0001	<0.0001
Main effect time^c^				0.04
Interaction effect^d^				0.25

^a^ Estimate represents the mean from a multivariate regression model for longitudinal measures.

^b^ Main effect preservation type represents the overall effect, regardless of the timepoint.

^c^ Main effect time represents estimates the changes over time in BDIS, regardless of preservation type.

^d^ Interaction effect investigate if the evolution of BDIS, over time differs between preservation types.

**FIGURE 3:**
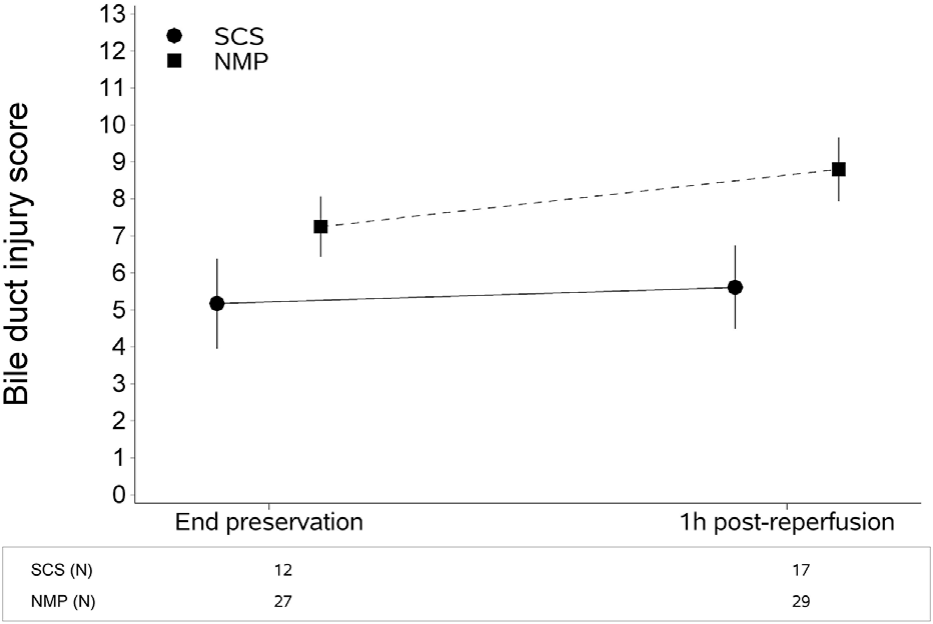
Continuous liver normothermic machine perfusion (NMP) is associated with higher bile duct injury score compared to static cold storage (SCS). This difference is significant both at the end of preservation and 1 hour after reperfusion. The plot shows estimates (mean from the multivariate regression model) with confidence intervals. The bile duct injury score was estimated with a multivariate regression model for longitudinal measures (Table 2).

**FIGURE 4:**
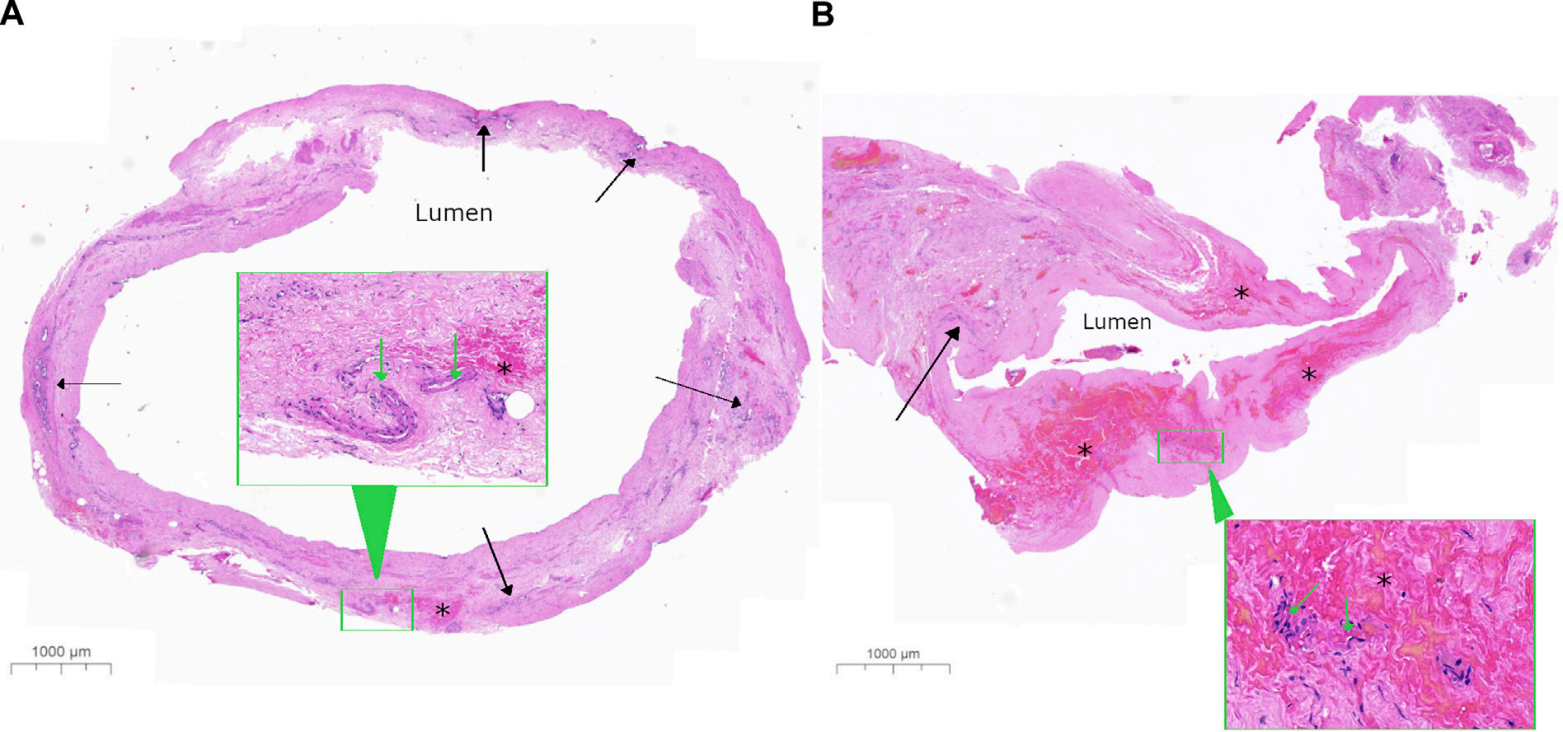
Representative histological image of bile ducts with low and high bile duct injury score. Low-power images show a complete transverse section from a bile duct with **(A)** a low bile duct injury score (BDIS) of 4 and **(B)** a high BDIS of 12. The stroma of the bile duct wall appears normal in **(A)** but appears amorphous pink with no nuclear detail in **(B)**. In **(A)**, the deep perinuclear glands (solid arrow) look relatively normal compared to **(B)** where only a few damaged peribiliary glands are visible with nuclear staining that is not readily identifiable as glands at this power. Additionally, there is focal interstitial haemorrhage (*) in **(A)** compared to extensive interstitial haemorrhage in **(B)**. The insets in both **(A,B)** show higher-power images of the areas outlined in a green rectangle to demonstrate that the arteries have good nuclear detail in **(A)** with less loss of nuclei in **(B)**, which are difficult to assess in this haemorrhagic area.

The type of liver preservation significantly affected the severity of BDIS, regardless of time (*p* < 0.001; Table 2). The overall BDIS estimate was significantly higher in the NMP group [8.02 (95% CI: 7.40, 8.65)] than in the SCS group [5.39 (95% CI: 4.52, 6.26), *p* < 0.0001]. This difference was significant both at the end of preservation [NMP: 7.25 (95% CI: 6.44, 8.06), SCS: 5.17 (95% CI: 3.96, 6.39); *p* = 0.006] and at 1-h post-reperfusion [NMP: 8.80 (95% CI: 7.94, 9.66), SCS: 5.61 (95% CI: 4.49, 6.73); *p* < 0.001] (Figure 3). There was no evidence that the size of BDIS change in time depends on preservation type (interaction effect, *p* = 0.25). Representative images of bile ducts with low or high BDIS are provided in Figure 4. A *post hoc* sensitivity analysis excluding the four transplants with BDIS improving after reperfusion confirmed these findings (Supplementary Table S2).

The NMP-preserved liver that developed IC after transplantation had a BDIS of 6 on a circumferential bile duct biopsy taken 1-h post-reperfusion. There were 15 other livers (6 NMP, 9 SCS) with a circumferential bile duct biopsy taken 1-h post-reperfusion with a BDIS >6 (range 7–12) that did not develop IC.

### Does the Effect of Type of Preservation Differs Among Donor Types?

There was no difference in BDIS estimate between DBD [7.1 (95% CI: 6.4, 7.9)] and DCD liver grafts [7.0 (95% CI: 5.9, 8.1), *p* = 0.87]. The BDIS estimate in NMP was higher than in SCS in both DBD and DCD transplants (Supplementary Table S3 and Supplementary Figure S4). There was no evidence that the effect of preservation type on histological injury of the bile duct differs between DBD and DCD (interaction effect, *p* = 0.989).

### Characteristics Influencing the Severity of BDIS

To investigate which factor influences the severity of bile duct injury, we used bivariate linear models with BDIS as endpoint, and tested donor, recipient, transplant, and perfusion characteristics (Table 3, Supplementary Table S4). Donor type and donor warm ischemic time did not affect BDIS. The log-transformed highest concentration of gamma glutamyl transferase in the donor and cold ischemic time showed significant associations with BDIS. Donor blood group also influenced the severity of bile duct injury, with AB group donors associated with the lowest BDIS. Additionally, donor history of smoking or alcohol intake and the use of dopamine correlated with histological injury severity. Recipient-related characteristics did not show associations with BDIS. Blood group mismatch, NMP duration, bile volume during perfusion, implantation time, and hemodynamic post-reperfusion did not impact BDIS. An additive multivariable model including significant variables from univariate analysis showed that time, type of preservation, and donor blood group independently influence bile duct injury as measured by BDIS (Table 3).

**TABLE 3:** Results from univariate and multivariate analysis exploring characteristics influencing the severity of the histological bile duct injury score.

Univariate analysis				
	Average of both timepoints			
	Beta (SE)	*p*-value	Mean (95%CI)	*p*-value
**Donor demographics**				
Donor type				0.87
DBD			7.1 (6.4; 7.9)	
DCD			7.0 (5.9; 8.1)	
Total donor warm ischemia time^a^ (min)	0.034 (0.056)	0.56		
Donor age	0.029 (0.019)	0.14		
Donor gender				0.98
Male			7.1 (6.3; 7.9)	
Female			7.1 (6.2; 8.0)	
Donor BMI	0.066 (0.060)	0.28		
Donor blood group				0.02
A			6.5 (5.8; 7.3)	
AB			4.5 (1.3; 7.6)	
B			8.9 (6.7; 11.1)	
0			7.9 (6.9; 8.8)	
Length of donor ICU stay	0.108 (0.144)	0.46		
Donor cause of death				0.16
Others			7.5 (5.8; 9.1)	
Trauma			4.9 (2.5; 7.3)	
Hypoxia			7.9 (6.6; 9.2)	
Cerebrovascular accident			6.9 (6.2; 7.7)	
DRI	0.154 (0.379)	0.69		
ET-DRI	0.898 (0.623)	0.16		
History of diabetes				0.63
No			7.2 (6.5; 7.8)	
Yes			6.7 (4.9; 8.5)	
History of smoking				0.02
No			7.6 (6.9; 8.3)	
Yes			6.2 (5.3; 7.2)	
History of alcohol consumption				0.02
No			7.4 (6.7; 8.0)	
Yes			5.5 (4.1; 7.0)	
History of cardiac disease				0.15
No			6.7 (6.0; 7.4)	
Yes			7.9 (6.4; 9.4)	
Vasopressor use				0.45
No			7.5 (6.6; 8.4)	
Yes			7.0 (6.3; 7.8)	
Dopamine				0.76
No			7.1 (6.3; 7.9)	
Yes			6.6 (4.0; 9.3)	
Dobutamine				0.34
No			7.0 (6.2; 7.8)	
Yes			8.6 (5.1; 12.2)	
Noradrenaline				0.56
No			7.4 (5.9; 9.0)	
Yes			6.9 (6.0; 7.8)	
Vasopressin				0.70
No			7.3 (6.0; 8.5)	
Yes			6.9 (5.9; 8.0)	
Highest AST	0.002 (0.008)	0.76		
Highest AST (log2)	0.449 (0.471)	0.35		
Highest ALT	−0.001 (0.006)	0.89		
Highest ALT (log2)	0.150 (0.250)	0.55		
Highest GGT	0.005 (0.003)	0.12		
Highest GGT (log2)	0.523 (0.231)	0.03		
Highest Sodium	−0.075 (0.039)	0.06		
Highest Bilirubin	0.011 (0.025)	0.67		
Highest Bilirubin (log2)	0.248 (0.171)	0.15		
Cold ischemia time (h)	−0.451 (0.112)	0.0001		
**Recipient demographics**				
Recipient age	−0.039 (0.027)	0.15		
Recipient gender				0.93
Male			7.1 (6.4; 7.8)	
Female			7.0 (6.0; 8.1)	
Recipient BMI	0.068 (0.053)	0.20		
Recipient blood group				0.06
A			6.5 (5.7; 7.3)	
AB			5.7 (3.4; 8.0)	
B			8.9 (6.9; 10.9)	
0			7.7 (6.7; 8.6)	
Blood group match				0.76
Identical			7.1 (6.4; 7.7)	
Compatible			7.5 (5.0; 9.9)	
Creatinine	0.006 (0.009)	0.50		
Bilirubin	0.004 (0.004)	0.37		
Recipient Bilirubin (log2)	0.296 (0.180)	0.11		
INR	0.007 (0.681)	0.99		
INR (log2)	−0.197 (0.838)	0.82		
Lab MELD	0.062 (0.053)	0.25		
**Transplantation**				
Steatosis				0.46
None			6.9 (5.8; 7.9)	
Mild			6.8 (5.8; 7.7)	
Moderate			7.9 (6.5; 9.3)	
Severe			8.0 (5.5; 10.5)	
Liver weight	0.001 (0.001)	0.17		
Duration NMP (min)	−0.002 (0.001)	0.054		
Volume Bile NMP (mL)	0.003 (0.004)	0.46		
Porto caval bypass				0.03
No			7.6 (6.8; 8.3)	
Yes			6.0 (4.7; 7.2)	
Veno-venous bypass				0.23
No			7.2 (6.6; 7.9)	
Yes			5.5 (2.4; 8.6)	
Vena cava anastomosis				0.04
Cava replacement			5.2 (3.4; 7.1)	
Piggyback			7.3 (6.7; 7.9)	
Portal vein anastomosis time (min)	−0.002 (0.018)	0.90		
Hepatic artery anastomosis time (min)	0.010 (0.016)	0.53		
Total implantation time (min)	0.004 (0.011)	0.75		
**Outcomes**				
Post-reperfusion syndrome^b^				0.28
No			7.3 (6.6; 7.9)	
Yes			6.5 (5.2; 7.7)	
Post-reperfusion Mean Arterial Pressure	0.029 (0.015)	0.06		
Post-reperfusion vasopressor				0.54
No			7.4 (6.6; 8.2)	
Yes			7.0 (6.2; 7.9)	
Post Reperfusion Lactate	0.118 (0.253)	0.64		
**Multivariate analysis** ^**c**^				
	**Beta (SE)**	***p*-value**		
Time		0.01		
End preservation	ref.			
1 h post-reperfusion	1.276 (0.480)			
Preservation type		0.002		
SCS	ref.			
NMP	3.828 (1.173)			
Cold ischemic time, h	0.256 (0.224)	0.26		
Donor highest serum GGT, log2	0.251 (0.198)	0.21		
Donor blood group		0.03		
A	−1.014 (0.577)	0.09		
AB	−3.463 (1.411)	0.02		
B	1.302 (1.185)	0.28		
0	ref.			
Donor history of smoking		0.29		
No	ref.			
Yes	−0.617 (0.577)			
Donor history of alcohol consumption		0.19		
No	ref.			
Yes	−0.961 (0.725)			

N.B the relationship between indication to transplantation and BDIS, could not be explored due to the small sample size in each individual indication.

The complete analysis is reported in Supplementary Table S4.

ALT, alanine transaminase; AST, aspartate transaminase; BMI, body mass index; DBD, donation after brain death; DCD, donation after circulatory arrest; DRI, donor risk index; ET-DRI, EuroTransplant donor risk index; GGT, gamma glutamyl transferase; INR, international normalized ratio; MELD, model for end-stage liver disease.

^a^ Total donor warm ischemic time in DCD, donors is measured from the withdraw of life sustaining therapy to cold flush.

^b^ Post-reperfusion syndrome was defined as > 30% drop in mean arterial pressure persisting for > 1 min within 5 min of reperfusion.

^c^ Result from an additive multivariable multivariate linear model for repeated measures, not considering interactions with time (due to sample size but also given the non-significant univariable results for these interaction terms). Dopamine use in the donor has been exclude as covariate because of excessive missing values. The model has been fitted on 75 observations.

## Discussion

In this study, we provide evidence of the differential impact of different liver preservation modalities on the histological injury to the extrahepatic bile duct. Using 85 biopsies collected during the COPE trial comparing 121 NMP to 101 SCS livers, we showed that the BDIS increases significantly over time, regardless of preservation modality or other donor or transplant characteristics. Interestingly, although continuous liver NMP was associated with significantly higher BDIS, this did not lead to higher incidence of IC in the COPE trial. Next to the effect of time and preservation type, donor blood group emerged as a factor that may influence the severity of histological bile duct injury.

Although the results of our study revealed significant differences in BDIS between NMP and SCS groups, the size of this difference (almost three points) should be placed in context. In this study, BDIS increased significantly over time, independently of the preservation strategy used. As the duration of preservation was longer in the NMP group (>12 h) than in the SCS group (5.75 h, Figure 1), the bile duct biopsies taken after preservation and 1-h post-reperfusion captured different stages of the increase of biliary injury severity over time in the two groups. Consequently, a direct comparison of biopsies after preservation and 1-h post-reperfusion between the two groups overestimates the effect of NMP on BDIS as it does not adjust for the additional damage caused by the longer preservation time in the NMP group. In other words, while the difference in BDIS estimates between NMP and SCS was 2.63 points (Table 2), the real magnitude of the contribution of NMP to BDIS increase may have been smaller since preservation times were inherently different in the two groups. Nonetheless, our results clearly showed that continuous liver NMP is independently associated with higher BDIS. The fact that a liver graft undergoes two hits of reperfusion during NMP (“on pump” and in the recipient) may explain the exacerbation of BDIS. However, the overestimation of the detrimental effect of liver NMP on BDIS may explain the comparable incidence of IC in the COPE trial despite significantly higher BDIS in the NMP group.

Additionally, our study raises concerns about the validity of BDIS as a surrogate endpoint of IC in perfused livers, and about currently defined cholangiocellular viability criteria. Three reports on bile duct histology during liver transplantation highlighted the univariate association between arteriolonecrosis, epithelial loss, stromal necrosis, injury to the peribiliary glands and the risk of IC after transplantation [3–5]. However, to the best of our knowledge no additional study has investigated whether these histological features remain independently associated with the risk of IC when adjusted for other well-known risk factors. In this study, we report that higher BDIS does not necessarily lead to increased IC after transplantation. This finding is in line with the observation that although more than 80% of liver grafts exhibit histological injury to the biliary epithelium at the end of SCS [4], only 10%–15% develop IC after transplantation, indicating that this injury can be recovered [15]. Therefore, determining first the contribution of bile duct histological injury to the overall risk of IC is crucial to elect BDIS as surrogate endpoint of this complication. Nevertheless, in a series of end-ischemic NMP of human livers, Matton et al. postulated that a BDIS score >4.75 points identifies high-risk grafts for IC, and cholangiocellular viability criteria based on bile biochemistry were developed to identify liver grafts likely to exceed this threshold [6]. The BDIS was calculated by adding up the score assigned to each histological alteration considered, implicitly assuming that they contribute to the risk of IC equally. However, the individual contribution of each histological lesion has never been investigated in multivariate analysis. Therefore, whether an additive BDIS is an accurate representation of biological events leading to IC remains unknown. Recently, de Jong et al. examined the histological aspect of the extrahepatic bile duct in livers transplanted after end-ischemic NMP [16]. They concluded that the currently defined cholangiocellular criteria correlate well with the histological damage, particularly of peribiliary glands and vascular plexus. These findings are not surprising as these criteria were specifically designed for this purpose, but they do not provide information on their accuracy since the study by de Jong et al. suffered from selection bias. Conversely, our study demonstrated that livers with BDIS scores considerably higher than the 4.75 threshold [6] can still have excellent outcomes. Indeed, while the NMP-preserved liver that developed IC displayed a BDIS score of 6 at 1 hour after reperfusion, 15 other grafts (9 SCS and 6 NMP) exhibited even higher BDIS scores (up to 12) at the same time point without developing IC. Although speculative, this observation, along with the absence of increased incidence of IC in the NMP group, suggests that BDIS overestimates the risk of IC in NMP-preserved livers. While we acknowledge that our findings may not be directly translatable to end-ischemic NMP, current cholangiocellular viability criteria may be too restrictive, potentially leading to unnecessary discarding livers that would remain free from IC.

This study also explored the factors influencing the BDIS. Time, preservation type, and donor blood group were found to be independent determinants of the score (Table 3). In this subset from the COPE liver NMP trial, DCD donors did not influence the BDIS. The results from the main trial showed that liver NMP exert a stronger protective influence on DCD grafts [9]. Considering that most DCD livers were preserved with NMP in this substudy, it is possible that liver NMP could have mitigated the adverse impact of DCDs on BDIS. However, due to the low number of DCD grafts in this substudy, the role of donor type on the severity of BDIS in livers preserved with NMP warrants further investigations. Cholangiocytes express ABO-antigens and a previous study by Sanchez-Urdazpal et al. reported increased biliary complications after ABO-incompatible transplants, possibly due to enhanced immunological damage [17]. However, donor blood group AB was associated with a significant reduction of BDIS in our study. We did not include ABO-incompatible transplant and blood group matching (identical vs. compatible) did not influence BDIS. Therefore, we have currently no explanation for the protective role of donor blood group AB. However, due to the small number of AB group grafts (*n* = 2) and the lack of correction for multiple testing, these results should be interpreted as exploratory.

This study has some limitations. Not all centres involved in the COPE trial participated in this substudy, and not all transplants were included, which may limit the generalizability of the results to the entire trial population. Excluded cases had a significantly longer cold ischemic time, a known risk factor for biliary injury [18]. However, it is unlikely that the small median difference in cold ischemic time (1.28 h, Supplementary Table S1) would have reversed the results. Due to sampling issues, most biopsies did not include the entire bile duct circumference. To strike a balance between sample size and reliable evaluation, only biopsies with at least half circumference were considered for the BDIS. Although misinterpretation of injury severity cannot be ruled out, there was no difference in the proportion of biopsies with at least half circumference between the two groups (Supplementary Table S5). Nevertheless, complete bile duct circumferences were evaluated for the NMP liver that developed IC and other 15 with higher BDIS, eliminating this risk. Moreover, this study emphasizes the need for improved standardization of sampling techniques in future studies investigating BDIS. It is crucial to sample an adequate length of the extrahepatic bile duct above the biliary cannula tip to increase the likelihood of obtaining a representative whole circumference sample. Nonetheless, our findings provide a strong rationale to reassess BDIS as a surrogate endpoint for IC and re-evaluate current cholangiocellular viability criteria accuracy.

In conclusion, histological bile duct injury worsens over time regardless of the preservation method. Continuous NMP is associated with higher BDIS, but the magnitude of this effect remains uncertain due to limitations inherent to machine perfusion trials logistic. However, the more severe histological injury during continuous NMP does not necessarily lead to increased IC. Therefore, BDIS may overestimate this risk in NMP-perfused livers, making it less suitable as a surrogate endpoint for cholangiocellular viability criteria definition. Understanding the biological significance of bile duct injury and cholangiocellular biology during liver NMP is crucial for improving donor liver risk assessment. Further investigations exploring the biological responses of cholangiocytes from different donor types to NMP could shed light on these intricate mechanisms. To this end, the evaluation of bio-banked liver tissue samples from the COPE trial using single-cell -omics studies is being considered, which holds promise for unravelling the complexities of bile duct injury and optimizing liver graft preservation and selection.

## Data Availability

The raw data supporting the conclusion of this article will be made available by the authors, without undue reservation.

## Abbreviations

BDIS, bile duct injury score; DBD, donation after brain death; DCD, donation after circulatory death; IC, ischemic cholangiopathy; NMP, normothermic machine perfusion; SCS, static cold storage.
