# Supplementary material for: The Effect of Continuous Liver Normothermic Machine Perfusion on the Severity of Histological Bile Duct Injury
Source: Transpl Int. 2023 Sep 4;36:11645. doi: 10.3389/ti.2023.11645 (PMC10505658; doi:10.3389/ti.2023.11645)
Supplement: Supplementary file 1 [file DataSheet1.pdf]

Supplementary Tables

Capsule sentence summary

A sub-study of the COPE liver NMP trial discovered that the bile duct injury score (BDIS) was higher during NMP than during SCS but did not lead to increased biliary complications. Therefore, BDIS may overestimate the risk of ischemic cholangiopathy after liver NMP.

Supplementary Table S1

Donor, recipient, and transplant characteristics of subjects excluded and included in the substudy of the COPE randomized controlled trial on continuous liver NMP investigating the effect of preservation type on BDIS.

	Excluded (n=157)	Included (n=65)	p-value
Donor demographics			
Donor Type, n (%)			0.09
DBD	123 (78.34%)	44 (67.69%)	
DCD	34 (21.66%)	21 (32.31%)	
Total donor warm ischemia time,[a] min	26 (22-30)	24 (21-28)	0.47
Donor age, years	55 (45-63)	58 (46-69)	0.34
Donor gender, n (%)			0.39
Male	92 (58.60%)	34 (52.31%)	
Female	65 (41.40%)	31 (47.69%)	
Donor BMI, Kg/m^2	26 (24-30)	25 (17-47)	0.01
Donor blood group, n (%)			0.38
A	68/157 (43.31%)	35/65 (53.85%)	
AB	6/157 (3.82%)	2/65 (3.08%)	
B	19/157 (12.10%)	5/65 (7.69%)	
0	64/157 (40.76%)	23/65 (35.38%)	
Donor admitted to the ICU, n (%)	153 (98.08%)	65 (100%)	0.26
Length of donor ICU stay, days	3 (2-5)	2 (2-4)	0.29
Donor cause of death, n (%)			0.23
Others	14/157 (8.92%)	8/65 (12.31%)	
Trauma	25/157 (15.92%)	4/65 (6.15%)	
Hypoxia	32/157 (20.38%)	13/65 (20.00%)	
Cerebrovascular accident	86/157 (54.78%)	40/65 (61.54%)	
DRI, points	1.43 (1.15-6.35)	1.53 (1.21-2.68)	0.16
ET-DRI, points	1.66 (1.46-1.95)	1.83 (1.54-2.17)	0.002
History of diabetes, n (%)	15/152 (9.87%)	6/65 (9.23%)	0.88
History of smoking, n (%)	55/156 (35.26%)	24/65 (36.92%)	0.81
History of alcohol consumption, n (%)	22/156 (14.10%)	11/64 (17.19%)	0.56
History of cardiac disease, n (%)	22/121 (18.18%)	10/56 (17.86%)	0.96
Vasopressors use, n (%)	111/155 (71.61%)	38/64 (59.38%)	0.08
Dopamine, n (%)	2/111 (1.80%)	3/38 (7.89%)	0.07
Dobutamine, n (%)	3/111 (2.70%)	2/38 (5.26%)	0.45
Noradrenaline, n (%)	88/111 (79.28%)	29/38 (76.32%)	0.70
Vasopressin, n (%)	46/106 (43.40%)	23/38 (60.53%)	0.07
Highest AST, IU/L	51 (26-103)	44 (26-66)	0.33
Highest ALT, IU/L	37 (22-83)	32 (19-57)	0.12

Highest GGT, IU/L	42 (23-90)	38 (24-115)	0.74
Highest bilirubin, µmol/L	8 (5-12)	8 (5-17)	0.09
Highest sodium, mEq/L	146 (140-153)	143 (139-149)	0.047
Cold ischemic time, hours	3.63 (2.08-7.38)	2.35 (2.00-5.38)	0.01
Duration NMP, min	530 (370-694)	587 (357-719)	0.57
Recipient demographics			
Transplant centre, n (%)			0.01
Cambridge	16 (10.19%)	8 (12.31%)	
King’s College	19 (12.10%)	6 (9.23%)	
Birmingham	66 (42.04%)	39 (60.00%)	
Royal Free	27 (17.20%)	7 (10.77%)	
Germany	7 (4.46%)	0 (0.00%)	
Spain	16 (10.19%)	0 (0.00%)	
Leuven	6 (3.82%)	5 (7.69%)	
Recipient age, years	55 (48-63)	56 (47-62)	0.87
Recipient gender, n (%)			0.25
Male	117 (74.52%)	43 (66.15%)	
Female	40 (25.48%)	22 (33.85%)	
Recipient BMI, kg/m^2	27 (24-32)	26 (24-30)	0.37
Recipient blood group, n (%)			0.82
A	72 (45.86%)	32 (49.23%)	
AB	7 (4.46%)	4 (6.15%)	
B	20 (12.74%)	6 (9.23%)	
0	58 (36.94%)	23 (35.38%)	
Blood group match, n (%)			1
Identical	9 (5.73%)	4 (6.15%)	
Compatible	148 (94.27%)	61 (93.85%)	
Creatinine, mmol/L	68 (49-95)	70 (55-93)	0.67
Bilirubin, µmol/L	26 (11-60)	27 (10-47)	0.99
INR	1.31 (1.20-1.50)	1.30 (1.20-1.60)	0.92
Lab MELD, points	14 (10-18)	12 (10-17)	0.29
Indication to transplantation, n (%)			0.64
Alcoholic cirrhosis	43 (27.39%)	22 (33.85%)	
Budd Chiari	5 (3.18%)	2 (3.08%)	
Caroli's syndrome	4 (2.55%)	1 (1.54%)	
Cryptogenic cirrhosis	8 (5.10%)	0 (0.00%)	
Hemochromatosis	20 (12.74%)	11 (16.92%)	
HAT	4 (2.55%)	2 (3.08%)	
Non-cirrhotic portal hypertension	1 (0.64%)	0 (0.00%)	
Ornithine transcarbamylase deficiency	4 (2.55%)	1 (1.54%)	
Polycystic liver disease	13 (8.28%)	4 (6.15%)	
Biliary cirrhosis	26 (16.56%)	6 (9.23%)	
Ischemic cholangiopathy	1 (0.64%)	0 (0.00%)	
Sarcoidosis	7 (4.46%)	6 (9.23%)	
Secondary sclerosing cholangitis	21 (13.38%)	10 (15.38%)	
Transplantation			
Steatosis, n (%)			0.37
None	58/157 (36.94%)	22/64 (34.38%)	
Mild	74/157 (47.13%)	26/64 (40.63%)	
Moderate	16/157 (10.19%)	12/64 (18.75%)	

Severe	9/157 (5.73%)	4/64 (6.25%)	
Liver weight, g	1565 (1326-1851)	1411 (1180-1658)	0.16
Porto caval bypass, n (%)	51/140 (36.43%)	16/58 (27.59%)	0.23
Veno-venous bypass, n (%)	9/142 (6.34%)	2/58 (3.45%)	0.42
Vena cava anastomosis, n (%)			0.15
Cava replacement	28/150 (18.67%)	7/65 (10.77%)	
Piggyback	122/150 (81.33%)	58/65 (89.23%)	
Portal vein anastomosis time, min	38 (29-49)	32 (26-44)	0.03
Hepatic artery anastomosis time, min	32 (25-44)	33 (26-40)	0.57
Total implantation time, min	72 (56-85)	64 (53-86)	0.25
Intra-operative immunosuppression, n (%)			0.07
None	59/157 (37.58%)	37/65 (56.92%)	
Others	11/157 (7.01%)	1/65 (1.54%)	
Methylprednisolone	84/157 (53.50%)	26/65 (40.00%)	
Methylprednisolone + others	1/157 (0.64%)	0/65 (0.00%)	
Methylprednisolone + tacrolimus + others	1/157 (0.64%)	0/65 (0.00%)	
Basiliximab + methyprednisolone	1/157 (0.64%)	0/65 (0.00%)	
Basiliximab + methyprednisolone + tacrolimus	0/157 (0.00%)	1/65 (1.54%)	
Outcomes			
Peak AST within 7d, IU/L	689 (306-1589)	497 (290-1145)	0.11
Peak ALT within 7d, IU/L	346 (57-924)	419 (214-1085)	0.14
Peak Bilirubin, µmol/L	57 (24-120)	70 (33-136)	0.19
Peak GGT, IU/L	494 (289-740)	556 (350-797)	0.23
Peak INR	1.60 (1.40-1.90)	1.70 (1.46-2.00)	0.18
Post-reperfusion syndrome,[b] n (%)	33/153 (21.57%)	14/65 (21.54%)	1
Post-reperfusion mean arterial pressure, mmHg	67 (58-79)	66 (57-78)	0.78
Post-reperfusion vasopressor, n (%)	105/149 (70.47%)	40/63 (63.49%)	0.32
Post-reperfusion lactate, mmol/L	3.60 (2.70-4.40)	3.60 (2.85-4.45)	0.92
1-year patient survival, % (95%CI)	96.10 (91.50-98.20)	93.70 (84.10-97.60)	0.49
1-year graft survival, % (95%CI)	94.30 (89.30-97.00)	90.80 (80.60-95.70)	0.12
Graft loss at 1-year, % (95%CI)	5.70 (2.80-10.10)	9.20 (3.70-17.80)	0.11

[a] Total donor warm ischemic time in DCD donors is measured from the withdraw of life sustaining therapy to cold flush.

[b] Post-reperfusion syndrome was defined as > 30% drop in mean arterial pressure persisting for > 1 min within five minutes of reperfusion.

ALT, alanine transaminase; AST, aspartate transaminase; BMI, body mass index; DBD, donation after brain death; DCD, donation after circulatory arrest; DRI, donor risk index; ET-DRI, EuroTransplant donor risk index; GGT, gamma glutamyl transferase; INR, international normalized ratio; MELD, model for end-stage liver disease.

Supplementary Table S2

Post-hoc sensitivity analysis considering only transplants with increasing bile duct injury score over time. Results are based on a multivariate regression model for longitudinal measures.

	SCS (n=21)	NMP (n=40)		
**BDIS**	**Estimate[a] (CI)**	**Estimate[a] (CI)**	**P-value**	**Bonferroni**
Main effect preservation type[b]	5.52 (4.57;6.46)	8.10 (7.44;8.75)	<.0001	.
End preservation	5.01 (3.62;6.40)	7.05 (6.17;7.94)	0.01	0.01
1h post-LT	6.02 (4.94;7.10)	9.14 (8.34;9.94)	<.0001	<.0001
Main effect time[c]				0.002
Interaction effect[d]				0.27

[a] Estimate represents the mean from a multivariate regression model for longitudinal measures.
[b] Main effect preservation type represents the overall effect, regardless of the timepoint.
[c] Main effect time represents estimates the changes over time in BDIS, regardless of preservation type.
[d] Interaction effect investigate if the evolution of BDIS over time differs between preservation types.

Supplementary Table S3

Multivariate regression models for longitudinal measures investigating the effect of preservation type on BDIS separately in DBD and DCD liver transplants.

**DBD liver transplants (n=44)**				
	**SCS (n=14)**	**NMP (n=30)**		
**BDIS**	**Estimate[a] (CI)**	**Estimate[a] (CI)**	**P-value**	**Bonferroni**
Main effect preservation type[b]	5.22 (3.99;6.44)	7.99 (7.17;8.81)	0.0005	.
End preservation	5.39 (3.85;6.94)	7.12 (6.05;8.18)	0.07	0.07
1h post-LT	5.04 (3.29;6.78)	8.85 (7.74;9.97)	0.0006	0.001
Main effect time[c]				0.30
Interaction effect[d]				0.12
**DCD liver transplants (n=21)**				
	**SCS (n=9)**	**NMP (n=12)**		
**BDIS**	**Estimate[a] (CI)**	**Estimate[a] (CI)**	**P-value**	**Bonferroni**
Main effect preservation type[b]	5.36 (4.16;6.56)	8.08 (7.16;9.01)	0.001	.
End preservation	4.50 (2.55;6.44)	7.57 (6.37;8.78)	0.01	0.02
1h post-LT	6.23 (5.05;7.41)	8.60 (7.34;9.85)	0.01	0.02
Main effect time[c]				0.05
Interaction effect[d]				0.59

Given the small size of these subgroups, these results are to be interpreted only as exploratory.
[a] Estimate represents the mean from a multivariate regression model for longitudinal measures.
[b] Main effect preservation type represents the overall effect, regardless of the timepoint.
[c] Main effect time represents estimates the changes over time in BDIS, regardless of preservation type.
[d] Interaction effect investigate if the evolution of BDIS over time differs between preservation types.

Supplementary Table S4

Results from univariate analysis exploring characteristics influencing the severity of the histological bile duct injury score at the end of preservation, 1 hour post-reperfusion, and averaged at both timepoints.

	End preservation				1 hour post-reperfusion				Average of both timepoints			
	**Beta (SE)**	**p-value**	**Mean (95%CI)**	**p-value**	**Beta (SE)**	**p-value**	**Mean (95%CI)**	**p-value**	**Beta (SE)**	**p-value**	**Mean (95%CI)**	**p-value**
Donor demographics												
Donor type					0.81			0.63				0.87
DBD			6.5 (5.6-7.3)				7.8 (6.8-8.8)				7.1 (6.4-7.9)	
DCD			6.7 (5.3-8.0)				7.4 (5.9-8.8)				7.0 (5.9-8.1)	
Total donor warm ischemia time[a] (min)									0.034 (0.056)	0.56		
Donor age	0.023 (0.023)	0.33			0.034 (0.026)	0.19			0.029 (0.019)	0.14		
Donor gender				0.72				0.73				0.98
Male			6.4 (5.4-7.4)				7.8 (6.6-8.9)				7.1 (6.3-7.9)	
Female			6.7 (5.6-7.7)				7.5 (6.3-8.7)				7.1 (6.2-8.0)	
Donor BMI	0.003 (0.062)	0.97			0.129 (0.088)	0.15			0.066 (0.060)	0.28		
Donor blood group				0.08				0.02				0.02
A			5.7 (4.8-6.6)				7.4 (6.4-8.3)				6.5 (5.8-7.3)	
AB			6.4 (2.3-10.6)				2.5 (0.0-6.1)				4.5 (1.3-7.6)	
B			8.3 (5.8-10.9)				9.5 (5.9-13.1)				8.9 (6.7-11.1)	
0			7.3 (6.2-8.5)				8.4 (7.1-9.7)				7.9 (6.9-8.8)	
Length of donor ICU stay	0.123 (0.187)	0.51			0.094 (0.193)	0.63			0.108 (0.144)	0.46		
Donor cause of death				0.35				0.17				0.16
Others			6.6 (4.6-8.6)				8.3 (6.3-10.4)				7.5 (5.8-9.1)	
Trauma			3.8 (0.7-6.9)				6.0 (2.9-9.1)				4.9 (2.5-7.3)	
Hypoxia			6.8 (5.3-8.2)				9.1 (7.3-10.9)				7.9 (6.6-9.2)	
Cerebrovascular accident			6.7 (5.8-7.6)				7.1 (6.1-8.2)				6.9 (6.2-7.7)	
DRI	0.261 (0.411)	0.53			0.047 (0.565)	0.94			0.154 (0.379)	0.69		
ET-DRI	1.049 (0.636)	0.11			0.747 (0.984)	0.45			0.898 (0.623)	0.16		
History of diabetes				0.15				0.71				0.63
No			6.8 (6.0-7.5)				7.6 (6.7-8.4)				7.2 (6.5-7.8)	
Yes			5.3 (3.5-7.2)				8.0 (5.6-10.5)				6.7 (4.9-8.5)	
History of smoking				0.051				0.12				0.02
No			7.1 (6.2-7.9)				8.1 (7.1-9.1)				7.6 (6.9-8.3)	

Yes			5.7 (4.5-6.8)				6.8 (5.4-8.1)				6.2 (5.3-7.2)	
History of alcohol consumption				0.15				0.04				0.02
No			6.8 (6.0-7.5)				8.0 (7.1-8.9)				7.4 (6.7-8.0)	
Yes			5.4 (3.8-7.1)				5.6 (3.6-7.7)				5.5 (4.1-7.0)	
History of cardiac disease				0.24				0.27				0.15
No			6.3 (5.5-7.2)				7.1 (6.1-8.1)				6.7 (6.0-7.4)	
Yes			7.5 (5.7-9.3)				8.3 (6.5-10.1)				7.9 (6.4-9.4)	
Vasopressor use				0.23				0.95				0.45
No			7.2 (6.1-8.3)				7.8 (6.6-9.0)				7.5 (6.6-8.4)	
Yes			6.4 (5.5-7.2)				7.7 (6.6-8.8)				7.0 (6.3-7.8)	
Dopamine				0.04				0.24				0.76
No			6.7 (5.8-7.6)				7.5 (6.3-8.7)				7.1 (6.3-7.9)	
Yes			3.4 (0.5-6.3)				9.9 (5.9-13.9)				6.6 (4.0-9.3)	
Dobutamine				0.56				0.14				0.34
No			6.5 (5.5-7.4)				7.5 (6.3-8.7)				7.0 (6.2-7.8)	
Yes			5.5 (2.3-8.7)				11.7 (6.2-17.2)				8.6 (5.1-12.2)	
Noradrenaline				0.57				0.74				0.56
No			6.9 (5.1-8.6)				8.0 (5.6-10.4)				7.4 (5.9-9.0)	
Yes			6.3 (5.2-7.4)				7.6 (6.2-9.0)				6.9 (6.0-7.8)	
Vasopressin				0.48				0.96				0.70
No			6.9 (5.3-8.4)				7.7 (5.9-9.4)				7.3 (6.0-8.5)	
Yes			6.2 (5.0-7.4)				7.7 (6.1-9.3)				6.9 (5.9-8.0)	
Highest AST	0.007 (0.007)	0.29			-0.002 (0.014)	0.86			0.002 (0.008)	0.76		
Highest AST (log2)	0.451 (0.479)	0.36			0.447 (0.659)	0.50			0.449 (0.471)	0.35		
Highest ALT	0.002 (0.003)	0.57			-0.003 (0.010)	0.74			-0.001 (0.006)	0.89		
Highest ALT (log2)	0.189 (0.287)	0.51			0.111 (0.366)	0.76			0.150 (0.250)	0.55		
Highest GGT	0.004 (0.003)	0.17			0.007 (0.006)	0.27			0.005 (0.003)	0.12		
Highest GGT (log2)	0.403 (0.269)	0.14			0.642 (0.342)	0.07			0.523 (0.231)	0.03		
Highest Sodium	-0.080 (0.051)	0.13			-0.070 (0.049)	0.16			-0.075 (0.039)	0.06		
Highest Bilirubin	0.007 (0.042)	0.88			0.015 (0.023)	0.52			0.011 (0.025)	0.67		
Highest Bilirubin (log2)	0.206 (0.222)	0.36			0.289 (0.210)	0.18			0.248 (0.171)	0.15		
Cold ischemia time (h)	-0.344 (0.147)	0.02			-0.558 (0.141)	0.0002			-0.451 (0.112)	0.0001		
Recipient demographics[b]												
Recipient age	-0.051 (0.032)	0.12			-0.026 (0.037)	0.48			-0.039 (0.027)	0.15		
Recipient gender				0.92				0.96				0.93

Male			6.6 (5.7-7.4)				7.7 (6.7-8.6)				7.1 (6.4-7.8)	
Female			6.5 (5.2-7.7)				7.6 (6.2-9.0)				7.0 (6.0-8.1)	
Recipient BMI	0.043 (0.055)	0.43			0.092 (0.078)	0.24			0.068 (0.053)	0.20		
Recipient blood group				0.05				0.09				0.06
A			5.7 (4.7-6.6)				7.4 (6.3-8.5)				6.5 (5.7-7.3)	
AB			7.1 (4.1-10.0)				4.3 (1.3-7.4)				5.7 (3.4-8.0)	
B			8.6 (6.4-10.7)				9.2 (6.2-12.2)				8.9 (6.9-10.9)	
0			7.2 (6.0-8.3)				8.2 (6.9-9.5)				7.7 (6.7-8.6)	
Blood group match				0.43				0.77				0.76
Identical			6.5 (5.7-7.2)				7.7 (6.8-8.5)				7.1 (6.4-7.7)	
Compatible			7.7 (4.6-10.9)				7.2 (4.0-10.4)				7.5 (5.0-9.9)	
Creatinine	0.005 (0.011)	0.64			0.007 (0.012)	0.53			0.006 (0.009)	0.50		
Bilirubin	0.007 (0.007)	0.32			0.001 (0.005)	0.86			0.004 (0.004)	0.37		
Bilirubin (log2)	0.316 (0.223)	0.16			0.277 (0.239)	0.25			0.296 (0.180)	0.11		
INR	0.419 (0.849)	0.62			-0.405 (0.853)	0.64			0.007 (0.681)	0.99		
INR (log2)	0.368 (1.032)	0.72			-0.762 (1.070)	0.48			-0.197 (0.838)	0.82		
Lab MELD	0.092 (0.063)	0.15			0.032 (0.071)	0.66			0.062 (0.053)	0.25		
Transplantation												
Steatosis				0.81				0.16				0.46
None			6.2 (4.7-7.6)				7.5 (6.2-8.9)				6.9 (5.8-7.9)	
Mild			6.6 (5.4-7.8)				7.0 (5.8-8.2)				6.8 (5.8-7.7)	
Moderate			6.9 (5.4-8.4)				9.0 (7.0-11.0)				7.9 (6.5-9.3)	
Severe			5.6 (2.9-8.3)				10.4 (6.7-14.1)				8.0 (5.5-10.5)	
Liver weight	0.001 (0.001)	0.56			0.002 (0.001)	0.12			0.001 (0.001)	0.17		
Duration NMP (min)	-0.003 (0.002)	0.17			-0.002 (0.002)	0.16			-0.002 (0.001)	0.054		
Volume Bile NMP (mL)	0.009 (0.005)	0.12			-0.002 (0.006)	0.74			0.003 (0.004)	0.46		
Porto caval bypass				0.001				0.58				0.03
No			7.1 (6.3-7.9)				8.0 (7.0-9.0)				7.6 (6.8-8.3)	
Yes			4.5 (3.1-5.8)				7.5 (5.8-9.1)				6.0 (4.7-7.2)	
Veno-venous bypass				0.34				0.38				0.23
No			6.7 (5.8-7.5)				7.8 (6.9-8.7)				7.2 (6.6-7.9)	
Yes			5.0 (1.6-8.4)				6.0 (2.0-10.0)				5.5 (2.4-8.6)	
Vena cava anastomosis				0.08				0.13				0.04
Cava replacement			4.4 (2.0-6.9)				6.0 (3.8-8.3)				5.2 (3.4-7.1)	
Piggyback			6.7 (6.0-7.5)				7.9 (7.0-8.7)				7.3 (6.7-7.9)	

Portal vein anastomosis time (min)	-0.005 (0.019)	0.81			0.000 (0.026)	1			-0.002 (0.018)	0.90		
Hepatic artery anastomosis time (min)	0.020 (0.016)	0.23			0.000 (0.024)	1			0.010 (0.016)	0.53		
Total implantation time (min)	0.009 (0.013)	0.46			-0.002 (0.016)	0.89			0.004 (0.011)	0.75		
Outcomes												
Post-reperfusion syndrome[c]				0.19				0.63				0.28
No			6.8 (6.0-7.6)				7.7 (6.8-8.7)				7.3 (6.6-7.9)	
Yes			5.7 (4.2-7.1)				7.3 (5.6-8.9)				6.5 (5.2-7.7)	
Post-reperfusion Mean Arterial Pressure	0.044 (0.017)	0.01			0.014 (0.021)	0.50			0.029 (0.015)	0.06		
Post-reperfusion vasopressor				0.03				0.42				0.54
No			7.6 (6.5-8.6)				8.2 (6.9-9.5)				7.4 (6.6-8.2)	
Yes			5.9 (5.0-6.8)				7.6 (6.6-8.6)				7.0 (6.2-7.9)	
Post Reperfusion Lactate	-0.002 (0.297)	01			0.238 (0.339)	0.49			0.118 (0.253)	0.64		

[a] Total donor warm ischemic time in DCD donors is measured from the withdraw of life sustaining therapy to cold flush.

[b] Although a model has been used for both timepoints, it is meaningful to interpret the relationship between recipients' characteristics and BDIS 1 hour post-reperfusion only.

[c] Post-reperfusion syndrome was defined as > 30% drop in mean arterial pressure persisting for > 1 min within five minutes of reperfusion.

ALT, alanine transaminase- AST, aspartate transaminase- BMI, body mass index- DBD, donation after brain death- DCD, donation after circulatory arrest- DRI, donor risk index- ET-DRI, EuroTransplant donor risk index- GGT, gamma glutamyl transferase- INR, international normalized ratio- MELD, model for end-stage liver disease.

Supplementary Table S5

Comparison of the completeness of the extra-hepatic bile duct biopsies between the two preservation groups.

	**SCS (n=101)**	**NMP (n=121)**	**p-value**
**Bile duct biopsy completeness**			0.06
≥ ½ bile duct circumference, n (%)	23 (29.57%)	42 (35.43%)	
< ½ bile duct circumference, n (%)	78 (71.43%)	79 (85.57%)	

Supplementary Figure S1

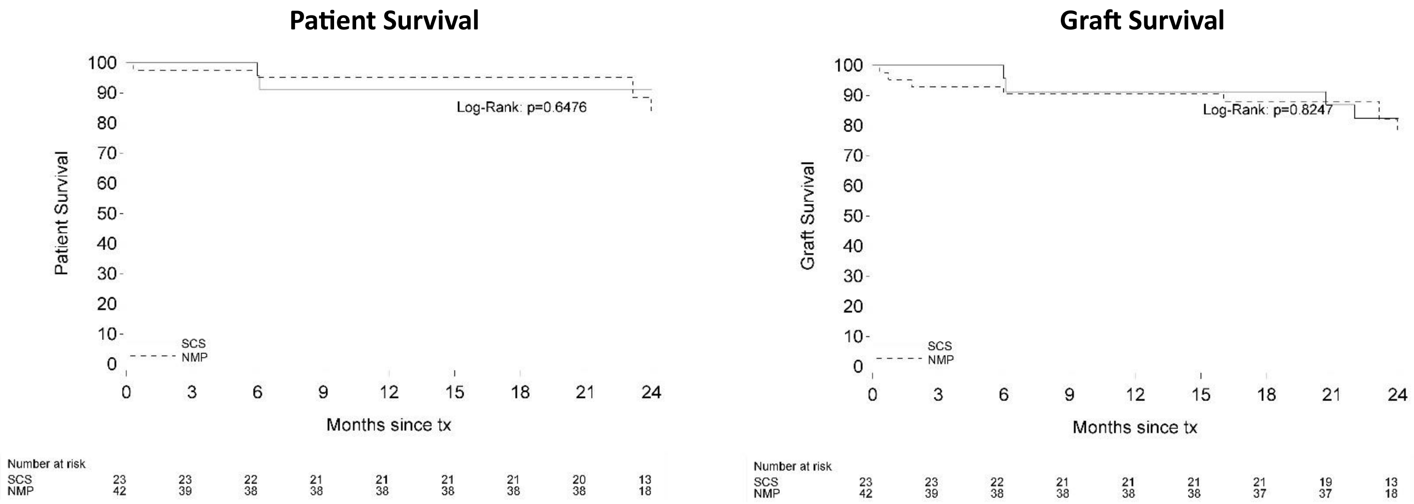

Figure S1. Kaplan-Meier curves estimating patient and graft survival of the transplants selected for this substudy of the COPE trial on liver NMP, showing no difference in 1 or 2-year survival between the two preservation groups.

Supplementary Figure S2

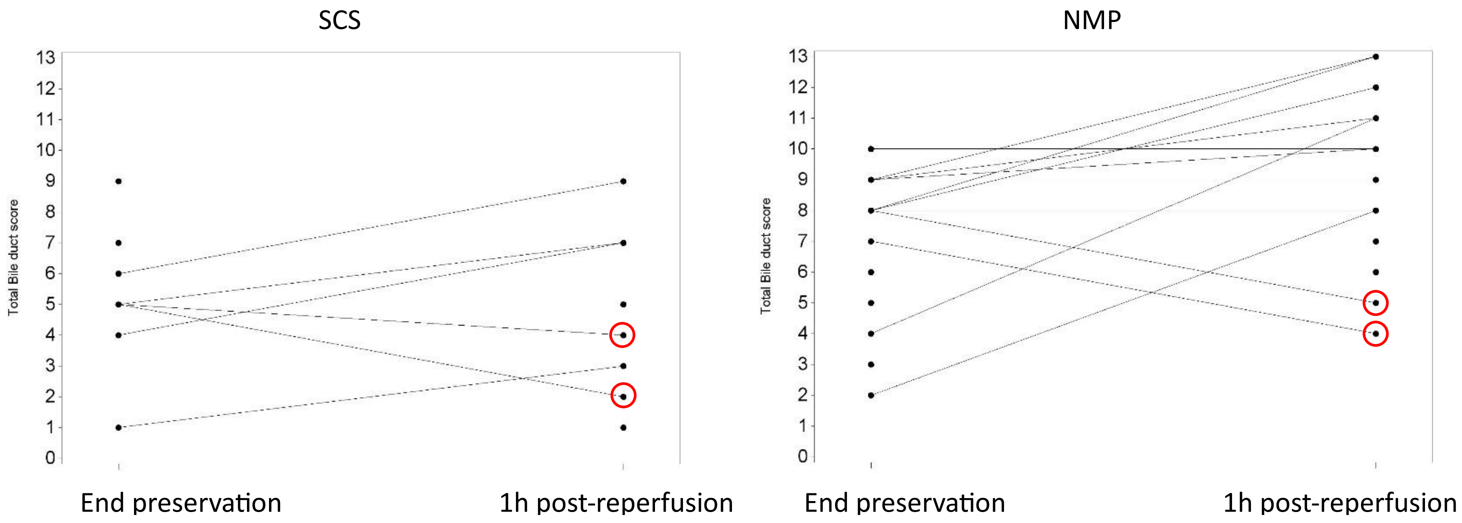

Figure S2. The bile duct injury score increased after graft reperfusion in most transplants, but in 4 livers equally distributed between the two groups it was found to be improved after 1-hour of reperfusion (red circles). Re-evaluation of these 2 biopsy pairs revealed signs of sampling injury in biopsies taken at the end of preservation. NMP, normothermic machine perfusion; SCS, static cold storage.

Supplementary Figure S3

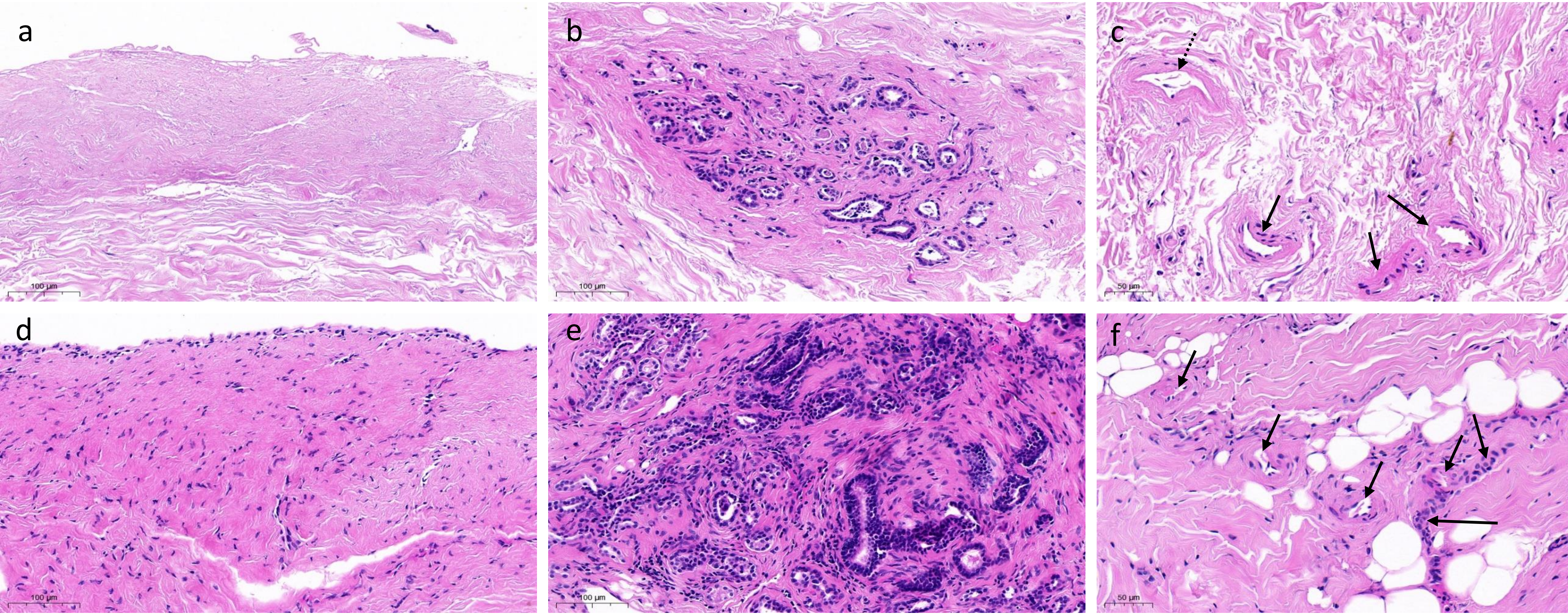

Figure S4. Representative histology of bile duct biopsies in transplants with improving BDIS after transplantation. a-c: bile duct biopsy at the end of preservation in a liver graft randomized to NMP preservation; d-f: bile duct biopsy 1-hour after transplantation of the same liver showing much more severe injury at the end of preservation indicative of surgical handling injury. Images are all taken at the same magnification. a&d: showing near complete loss of stromal nuclei (nuclei stain blue) in the end of preservation biopsy with plentiful nuclei in the 1-hour post reperfusion biopsy. b&e: moderate damage to the peribiliary glands in the end of preservation biopsy with minimal damage in the 1-hour post-reperfusion biopsy. c&f: there is more nuclear loss (nuclei stain blue) in the arteries (arrows) in the end of preservation biopsy than the 1-hour post-transplant biopsy. The most severely injured arteries have a dotted arrow.

Supplementary Figure S4

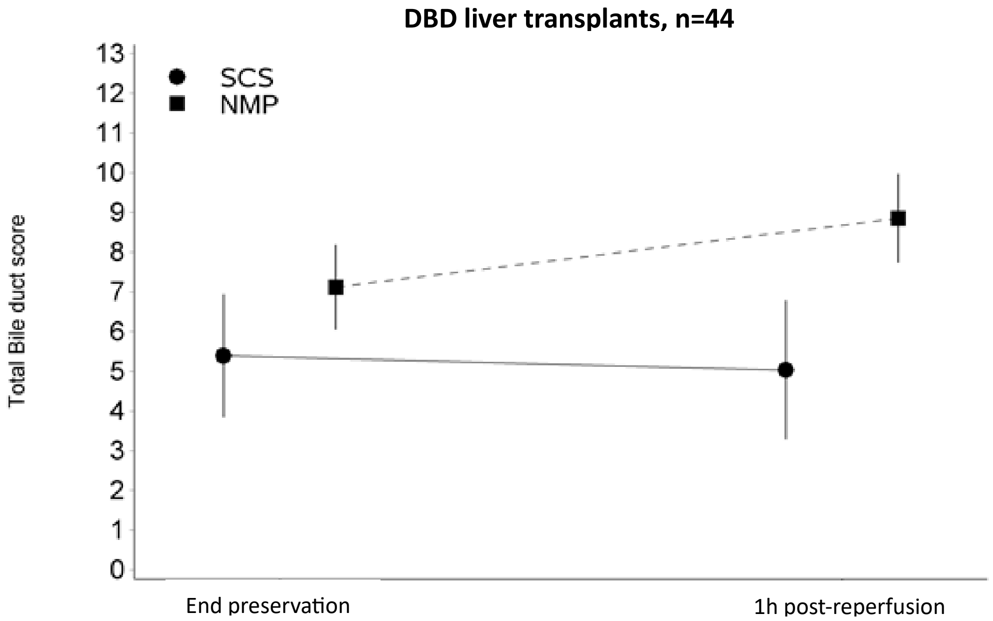

	End preservation	1h post-reperfusion
SCS (N)	9	9
NMP (N)	19	22

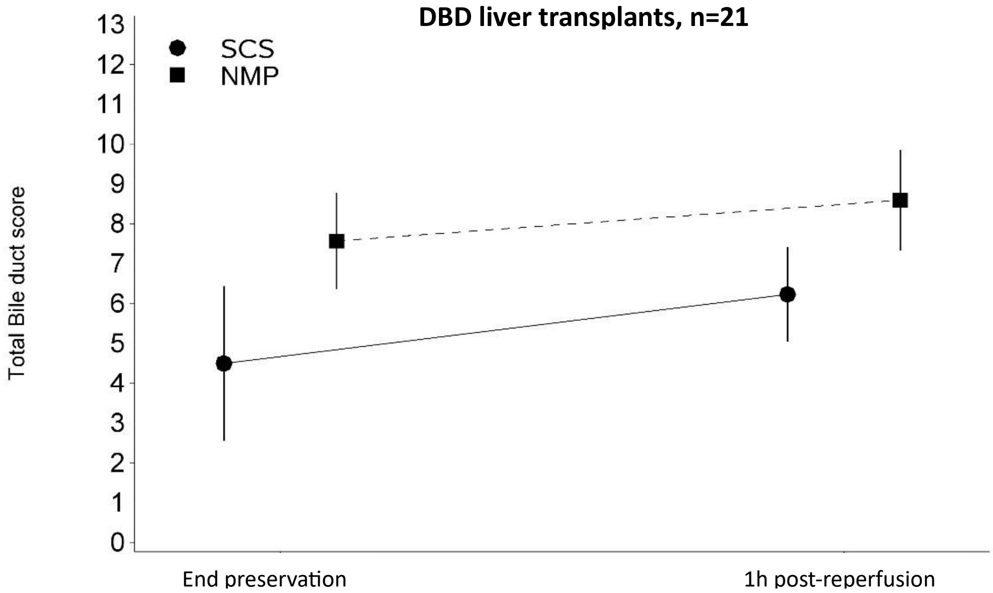

	End preservation	1h post-reperfusion
SCS (N)	3	8
NMP (N)	8	7

Figure S4. Continuous liver normothermic machine perfusion preservation was associated with higher bile duct injury score at histology in both donation after brain death and donation after circulatory death liver transplantation. Given the small size of these subgroups these results are to be interpreted only as descriptive. DBD, donation after brain death; DCD, donation after circulatory death; NMP, normothermic machine perfusion; SCS, static cold storage.
